# Correction: A new parrot taxon from the Yucatán Peninsula, Mexico—its position within genus *Amazona* based on morphology and molecular phylogeny

**DOI:** 10.7717/peerj.3475/correction-1

**Published:** 2017-09-12

**Authors:** Tony Silva, Antonio Guzmán, Adam D. Urantówka, Paweł Mackiewicz

**Affiliations:** 1 Miami, FL, United States of America; 2 Laboratorio de Ornitología, Facultad de Ciencias Biológicas, Universidad Autónoma de Nuevo León, Nuevo León, Mexico; 3 Department of Genetics, Wroclaw University of Environmental and Life Sciences, Wroclaw, Poland; 4 Faculty of Biotechnology, University of Wrocław, Wrocław, Poland

Tony Silva is a private researcher based in Miami, Florida. He is a member of the IFAS/TREC Advisory Committee at the University of Florida in Miami, however, his work on this research project was not sponsored by the university.

The full description of how collections were accessed are clarified in this version of the article.

