# Supplementary figures and images for: A new parrot taxon from the Yucatán Peninsula, Mexico—its position within genus Amazona based on morphology and molecular phylogeny
Source: PeerJ. 2017 Jun 27;5:e3475. doi: 10.7717/peerj.3475 (PMC5490482; doi:10.7717/peerj.3475)

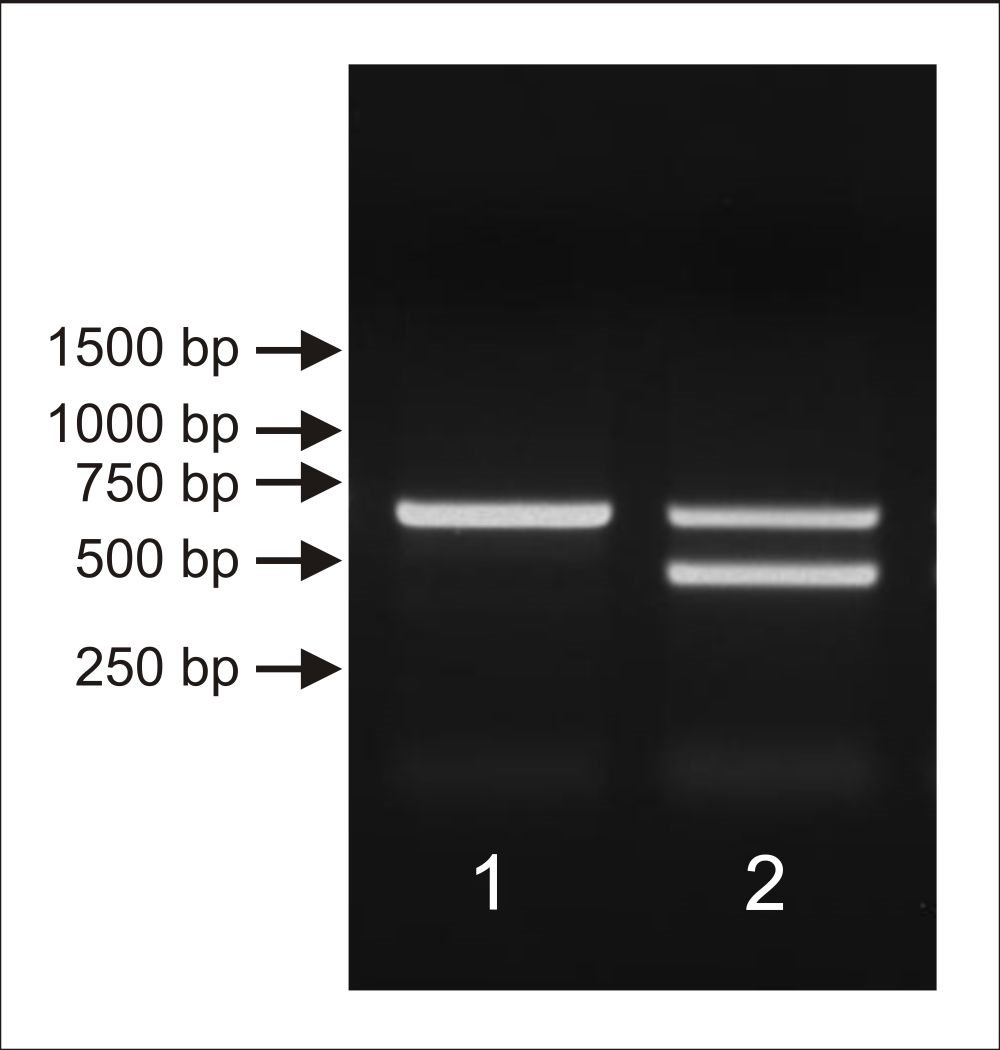

Supplement: Figure S1 — PCR products were electrophoresed on a 1% agarose. Positions of molecular weight marker bands are shown on the left using arrows. The results of DNA analysis confirmed that these specimens represent different sexes. Only one product with the length of approximately 600 bp was visible when template isolated from male holotype (1) was used in PCR reaction. Differently, two distinct bands with the length of approximately 600 bp (CHD-Z) and 480 bp for (CHD-W) were noticeable when DNA isolated from female allotype sample (2) was used in the test. [file peerj-05-3475-s003.jpg]

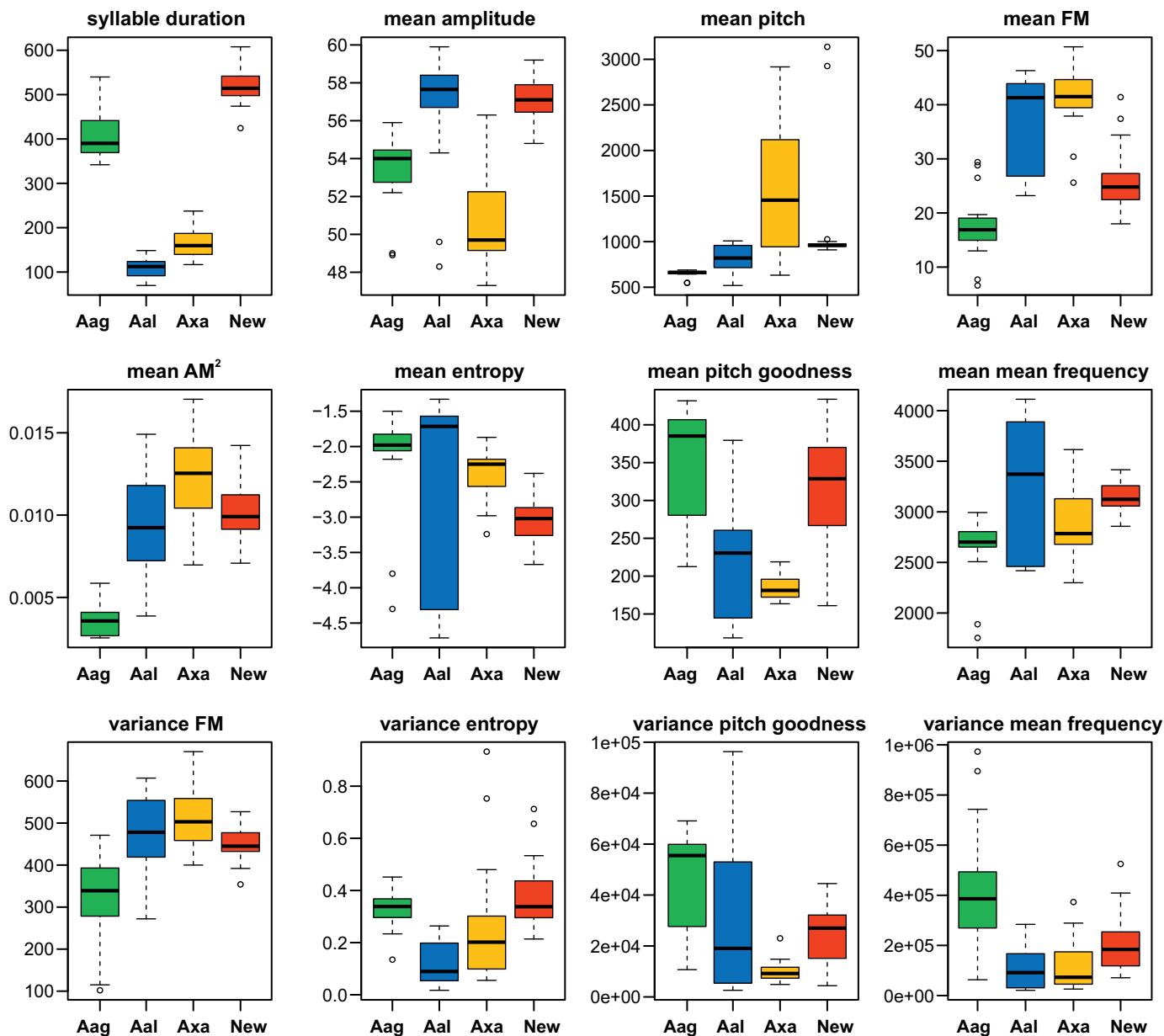

Supplement: Figure S2 — The thick line indicates median, the grey box shows quartile range and the whiskers denote the range without outliers. [file peerj-05-3475-s004.pdf]

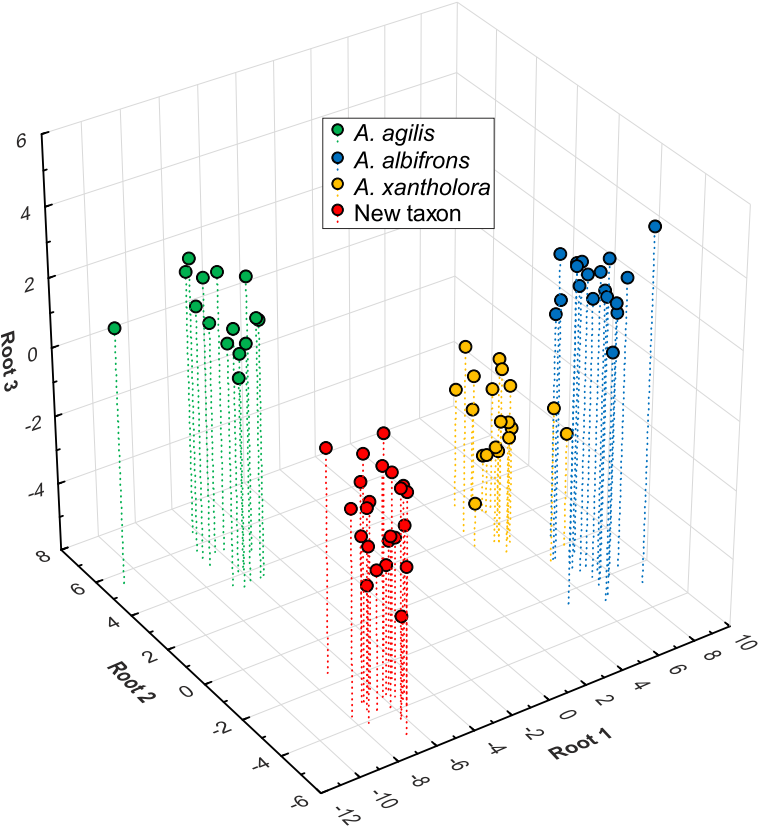

Supplement: Figure S3 [file peerj-05-3475-s005.pdf]
