# Supplementary material for: A new parrot taxon from the Yucatán Peninsula, Mexico—its position within genus Amazona based on morphology and molecular phylogeny
Source: PeerJ. 2017 Jun 27;5:e3475. doi: 10.7717/peerj.3475 (PMC5490482; doi:10.7717/peerj.3475)
Supplement: Table S1 [file peerj-05-3475-s006.pdf]

Table S1. GenBank accession numbers for three markers used in this study.

Taxon name	12S rRNA	16S rRNA	COI
Amazona aestiva aestiva	AY301328.1	AY301376.1	AY301424.1
Amazona aestiva xanthopteryx	AY301329.1	AY301377.1	AY301425.1
Amazona agilis	AY301330.1	AY301378.1	AY301426.1
Amazona albifrons albifrons	AY301331.1	AY301379.1	AY301427.1
Amazona albifrons nana	AY301332.1	AY301380.1	AY301428.1
Amazona albifrons saltuensis	AY301333.1	AY301381.1	AY301429.1
Amazona amazonica	AY301334.1	AY301382.1	AY301430.1
Amazona arausiaca	AY301335.1	AY301383.1	AY301431.1
Amazona auropalliata auropalliata	AY301336.1	AY301384.1	AY301432.1
Amazona auropalliata parvipes	AY301337.1	AY301385.1	AY301433.1
Amazona autumnalis autumnalis	AY301338.1	AY301386.1	AY301434.1
Amazona autumnalis lilacina	AY301339.1	AY301387.1	AY301435.1
Amazona barbadensis barbadensis	JX524615.1	JX524615.1	JX524615.1
Amazona brasiliensis	AY301341.1	AY301389.1	AY301437.1
Amazona collaria	AY301342.1	AY301390.1	AY301438.1
Amazona dufresniana	AY301343.1	AY301391.1	AY301439.1
Amazona farinosa farinosa	AY301344.1	AY301392.1	AY301440.1
Amazona farinosa guatemalae	AY301345.1	AY301393.1	AY301441.1
Amazona farinosa inornata	AY301346.1	AY301394.1	AY301442.1
Amazona farinosa virenticeps	AY301347.1	AY301395.1	AY301443.1
Amazona festiva bodini	AY301348.1	AY301396.1	AY301444.1
Amazona finschi	AY301349.1	AY301397.1	AY301445.1
Amazona guildingii	AY301350.1	AY301398.1	AY301446.1
Amazona imperialis	AY301351.1	AY301399.1	AY301447.1
Amazona kawalli	AY301352.1	AY301400.1	AY301448.1
Amazona leucocephala leucocephala	AY301353.1	AY301401.1	AY301449.1
Amazona ochrocephala nattereri	AY301354.1	AY301402.1	AY301450.1
Amazona ochrocephala ochrocephala	KM611467.1	KM611467.1	KM611467.1
Amazona ochrocephala ochrocephala	AY301355.1	AY301403.1	AY301451.1
Amazona ochrocephala panamensis	AY301356.1	AY301404.1	AY301452.1
Amazona ochrocephala xantholaema	AY301357.1	AY301405.1	AY301453.1
Amazona oratrix belizensis	AY301358.1	AY301406.1	AY301454.1
Amazona oratrix hondurensis	AY301359.1	AY301407.1	AY301455.1
Amazona oratrix oratrix	AY301360.1	AY301408.1	AY301456.1
Amazona pretrei	AY301361.1	AY301409.1	AY301457.1
Amazona rhodocorytha	AY301362.1	AY301410.1	AY301458.1
Amazona tucumana	AY301363.1	AY301411.1	AY301459.1
Amazona ventralis	AY301364.1	AY301412.1	AY301460.1
Amazona versicolor	AY301365.1	AY301413.1	AY301461.1
Amazona vinacea	AY301366.1	AY301414.1	AY301462.1
Amazona viridigenalis	AY301367.1	AY301415.1	AY301463.1
Amazona vittata	AY301368.1	AY301416.1	AY301464.1
Pionus menstruus	AY301373.1	AY301421.1	AY301469.1
