# Supplementary material for: A new parrot taxon from the Yucatán Peninsula, Mexico—its position within genus Amazona based on morphology and molecular phylogeny
Source: PeerJ. 2017 Jun 27;5:e3475. doi: 10.7717/peerj.3475 (PMC5490482; doi:10.7717/peerj.3475)
Supplement: Table S2 [file peerj-05-3475-s007.pdf]

Table S2. Partitioning schemes and nucleotide substitution models applied in the four algorithms for phylogenetic reconstruction.

<table>
<tr><th>Partition</th><th>MrBayes</th><th>Beast</th><th>TreeFinder</th><th>RAxML</th></tr>
<tr><td>12S rRNA
16S rRNA</td><td>mixed+I+Γ</td><td>TrN+I+Γ</td><td>TN+Γ</td><td rowspan="2">GTR+Γ</td></tr>
<tr><td>1 codon position of COI</td><td>mixed+I</td><td>TrNef+I</td><td>TN+Γ</td></tr>
<tr><td>2 codon position of COI</td><td>mixed</td><td>HKY</td><td>HKY {Ts=0.3, Tv=0.1}</td><td>GTR+Γ</td></tr>
<tr><td>3 codon position of COI</td><td>mixed+Γ</td><td>TrN+Γ</td><td>J1+Γ</td><td>GTR+Γ</td></tr>
</table>
