# Supplementary material for: A new parrot taxon from the Yucatán Peninsula, Mexico—its position within genus Amazona based on morphology and molecular phylogeny
Source: PeerJ. 2017 Jun 27;5:e3475. doi: 10.7717/peerj.3475 (PMC5490482; doi:10.7717/peerj.3475)
Supplement: Table S3 [file peerj-05-3475-s008.pdf]

Table S3. Values of parameters and the Akaike information criterion (AIC) for different diversification models fitted to the time-calibrated phylogeny.

Model description	Model name	Parameters	AIC	ΔAIC
Constant-rate models	Pure birth (Yule)	λ=0.492	-101.68	0.99
	Constant-rate birth–death (BD)	λ=0.276<br>μ=0.639	-102.52	0.15
Variable-rate models assuming different speciation rates within specified time intervals	Yule2rate	λ1=0.484<br>t1=0.060<br>λ2=0.750	-100.93	1.74
	Yule3rate	λ1=0.567<br>t1=2.828<br>λ2=0.195<br>t2=1.305<br>λ3=0.644	-101.55	1.12
	Yule4rate	λ1=0.138<br>t1=5.099<br>λ2=0.934<br>t2=3.199<br>λ3=0.229<br>t3=1.305<br>λ4=0.644	-102.67	0.00
	Yule4rate	λ1=0.138<br>t1=5.099<br>λ2=0.934<br>t2=3.199<br>λ3=0.274<br>t3=1.024<br>λ4=5.511<br>t4=1.017<br>λ5=0.642	-100.22	2.45
Variable-rate model assuming decreasing diversification through time in relation to increasing diversity with exponential fitting	DDX	λ=0.264<br>x=-0.219	-100.92	1.75
Variable-rate model assuming decreasing diversification through time in relation to increasing diversity with logistic fitting	DDL	λ=0.492<br>k=799068	-99.68	2.99
Variable-rate model assuming decreasing diversification through time caused by decreasing speciation rate	SPVAR	λ=0.770<br>μ=0.491<br>k=0.001	-100.51	2.16
Variable-rate model assuming decreasing diversification through time caused by increasing extinction rate	EXVAR	λ=0.763<br>μ=0.487<br>z=383.01	-100.52	2.15
Variable-rate model assuming decreasing diversification through time caused by decreasing speciation rate and increasing extinction rate	BOTHVAR	λ=0.770<br>μ=0.491<br>k=0.001<br>z=384.43	-98.51	4.16

λ, speciation; μ, extinction; t, time of rate-shift in millions of years ago; x, parameter controlling the magnitude of the rate change; K, parameter analogous to the ‘carrying capacity’ parameter in population ecology; k, parameter of the exponential change in speciation rate; z, parameter of the exponential change in extinction rate.
